# Bariatric Surgery and New-Onset Substance Use Disorders: A Systematic review and Meta-analysis

**DOI:** 10.1007/s11695-024-07130-7

**Published:** 2024-03-02

**Authors:** Silvia Martinelli, Niccolò Petrucciani, Luca Regazzi, Maria Rosaria Gualano

**Affiliations:** 1 Department of Life Sciences and Public Health, Università Cattolica del Sacro Cuore, Rome, Italy; 2 Department of Medical and Surgical Sciences and Translational Medicine, Division of General and Hepatobiliary Surgery, St. Andrea Hospital, Sapienza University of Rome, Via Di Grottarossa 1035-9, 00189 Rome, Italy; 3 Unicamillus - Saint Camillus International University of Health and Medical Sciences, Rome, Italy

**Keywords:** Bariatric Surgery, Obesity, Substance Use, New-onset Substance Use Disorder

## Abstract

Increasing evidence suggests that bariatric surgery (BS) patients are at risk for substance abuse disorders (SUD). The purpose of this systematic review and meta-analysis was to determine the relationship between BS and the development of new-onset substance abuse disorder (SUDNO) in bariatric patients. On October 31, 2023, we reviewed the scientific literature following PRISMA guidelines. A total of 3242 studies were analyzed, 7 met the inclusion criteria. The pooled incidence of SUDNO was 4.28%. Patients’ characteristics associated with SUDNO included preoperative mental disorders, high pre-BS BMI, and public health insurance. Surgical factors associated with new SUDNOs included severe complications in the peri- or postoperative period. The occurrence of SUDNOs is a non-negligeable complication after BS. Predisposing factors may be identified and preventive actions undertaken.

## Introduction

More than 1 billion people worldwide are obese and this number is still increasing. WHO estimates that by 2025, approximately 167 million people will become less healthy because they are overweight or obese and by 2030 48.9% of US population will be affected by obesity [1, 2]. Recent literature has pointed out the role of addiction mechanisms in the pathogenesis of obesity: reward dysfunction, impulsivity and emotion dysregulation [3, 4]. Reward dysfunction is mainly based on abnormalities in dopaminergic neurotransmission and increased activation of the dorsal and ventral striatum and orbitofrontal cortex in the presence of palatable food [3, 4]. Impulsivity, another common aspect of obesity and addiction disorders is the result of an executive control deficit that favors short-term food/drug gratifications instead of long-term benefits and correlates with reduced activation of the medial prefrontal cortex and other executive control regions [3, 4]. Overeating behaviors, such as drug use, may be triggered by emotional dysregulation with the consumption of high-fat and/or refined carbohydrate foods in response to emotional states such as stress or negative mood, determinants of food addiction and obesity [3, 4].

Currently, bariatric surgery (BS) is the most effective long-term treatment for patients with severe obesity, both in terms of weight loss and resolution of related comorbidities [5]. However, due to the mechanisms mentioned above, addictive behaviors may occur after bariatric surgery, including alcohol or substance use disorders [6, 7]. In addition, changes in the mesolimbic system after BS can lead to maladaptive reward-seeking behaviors, predisposing patients to the substance abuse [8, 9]. Substance addiction after BS can be problematic because of the particular physiology and metabolism of psychoactive substances [10]. Restrictive and malabsorptive BS techniques result in reduced stomach volume and modified intestinal absorption, quadrupling substance absorption [10–12]. This predisposes patients to an increased risk of intoxication or withdrawal, up to lethal overdose [13, 14].

The scientific literature on the incidence of new onset of substance use disorders (SUDNO), other than alcohol, after BS, is scarce, with only a few published series [8, 15–18]. The purpose of this study was to determine the incidence of SUDNO after BS, extensively reviewing the literature in order to assess the magnitude of this problem. Furthermore, the role of patient characteristics and surgery-related factors will be analyzed, in the effort to highlight the risk factors for SUDNO. Considering the physiology of the patient undergoing bariatric surgery, the identification and characterization of SUDNOs after BS will be important to guide the development of future guidelines and clinical actions to prevent addictive behaviors in this target patient population.

## Materials and Methods

### Study Design

A systematic review and proportion meta-analysis were conducted in keeping with the PRISMA guidelines [19]. The study was registered on PROSPERO database. The included population consisted of subjects ≥ 18 years old who have undergone elective bariatric surgery. Outcomes of interest included patient and surgical characteristics that were evaluated as risk factors for SUDNO. We defined our study eligibility using the populations-interventions-comparators-outcomes study design (PICO) framework.

- Population: Patients with obesity undergoing bariatric surgery
- Interventions: Bariatic surgery
- Outcomes: Incidence of SUDNO, excluding alcohol addiction, per the Diagnostic and Statistical Manual of Mental Disorders (DSM-5), International Classification of Diseases 10th Revision (ICD-10), or other criteria.

### Search Strategies

An electronic search of the in MEDLINE, Scopus, and Cochrane Library (Wiley) databases was performed on October 31, 2023, looking for relevant studies that could be included in this study. The search was performed by setting the following terms: “bariatric surgery [MeSH terms]” AND “substance use [MeSH terms]” OR “substance abuse [MeSH terms]” OR “cocaine use [MeSH terms]” OR “tobacco use [MeSH terms]” OR “caffeine use [MeSH terms]” OR “hallucinogens use [MeSH terms]” OR “volatile solvents use [MeSH terms]” OR “cannabinoid use [MeSH terms]” OR “hypnotics use [MeSH terms]” OR “opioids use [MeSH terms].” The same searches were carried out without using the function [MeSH terms] in order the extract the larger potential number of relevant articles. The Boolean operator “AND” was used to combine parts of the subject terms and “OR” was used to expand the search.

Two independent reviewers (SM and NP) screened titles and abstracts, assessed full-text versions, and extracted data. Disagreements were resolved by re-extraction or third-party adjudication.

### Data Extraction and Quality Assessment

The literature search was performed by two independent reviewers (SM and NP) using a predefined search strategy. No automation tools were used in the process. Duplicate studies were removed manually. Each reviewer then independently examined the titles, abstracts, and/or full texts of included manuscripts to ensure that all inclusion criteria were met before extracting the following data: [1] first author’s name, [2] year of publication, [3] study design, [4] country of origin, [5] number of patients included, [6] number of patients undergoing bariatric surgery who subsequently developed SUDNO, [7] number of patients undergoing each type of bariatric surgery who subsequently developed SUDNO, [8] number of patients undergoing bariatric surgery broken down by each type of SUDNO developed post-intervention, [9] number of patients undergoing bariatric surgery who did not develop SUDNO [10], patients’ characteristics [11], outcomes after bariatric surgery [12]. Missing or unclear information was not taken into consideration.

### Inclusion and Exclusion Criteria

Studies evaluating the impact of bariatric surgery on the development of SUDNO, on patients ≥ 18 years of age, that enrolled more than 10 patients, were included. Studies meeting any of the following exclusion criteria were excluded from the present review: [1] non-English studies, [2] animal studies, [3] abstracts, [4] review articles, [5] case reports or case series including less than 10 subjects; [6] editorials or letters, [7] studies not evaluating the impact of bariatric surgery on SUDNOs risk; [8] studies evaluating the impact of bariatric surgery on alcohol abuse risk, [9] studies including patients with previous substance use disorder, already present before bariatric surgery and object of the study, [10] patient age < 18. Where overlapping registries were identified or suspected, the more study was included for analysis.

### Primary and Secondary Outcomes

The primary outcome of the present study was to estimate the incidence of SUDNO, defined as new substance use, different from alcohol use, following bariatric surgery, using ICD-10 diagnostic criteria.

Secondary outcomes included preoperative and postoperative patients and surgical factors associated with SUDNO. Collected patient’s factors were age, gender, preoperative BMI, psychiatric comorbidities, university degree, and type of surgery. Surgical outcomes and collected risk factors were postoperative complications and type of bariatric surgical procedure. Primary outcome was defined at the time of the first studies’ selection, while secondary outcomes were included following title and abstract review in order to capture a complete and accurate representation of the patient and surgical characteristics that have been evaluated by current literature.

### Risk of Bias Assessment

The Risk of Bias In Non-randomized Studies of Interventions (ROBINS-I) tool was used to rate risk of bias for non-randomized included studies [20]. Two reviewers independently assessed each study (SM, NP) and disagreements were resolved by third-party adjudication (MRG). No automation tools were used.

This tool assesses seven domains: risk of bias from confounding, selection of participants, classification of interventions, deviations from intended interventions, missing data, measurement of outcomes, and selection of the reported results [20]. A proposed judgment about the risk of bias arising from each domain is generated by an algorithm, based on answers to the signaling questions. Judgment can be “Low,” “Moderate,” or “High” risk of bias, or can express “Some concerns” [20].

### Statistical Analysis

Patients’ characteristics and outcomes were summarized and described as means or medians for continuous variables or percentages for categorical variables. Meta-analysis was used to calculate a pooled SUDNO incidence as our study’s primary outcome. Pooled incidence was calculated using MEDCALC [21] and reported with 95% confidence intervals and I2 as a measure of heterogeneity. Heterogeneity was quantified by the I2 statistic as low < 50%, moderate 50–75%, or high > 75% [22]. Secondary outcomes were qualitatively analyzed due to heterogeneity of outcome reporting in the included studies, and tabulated.

## Results

### Study Selection

**Fig. 1:**
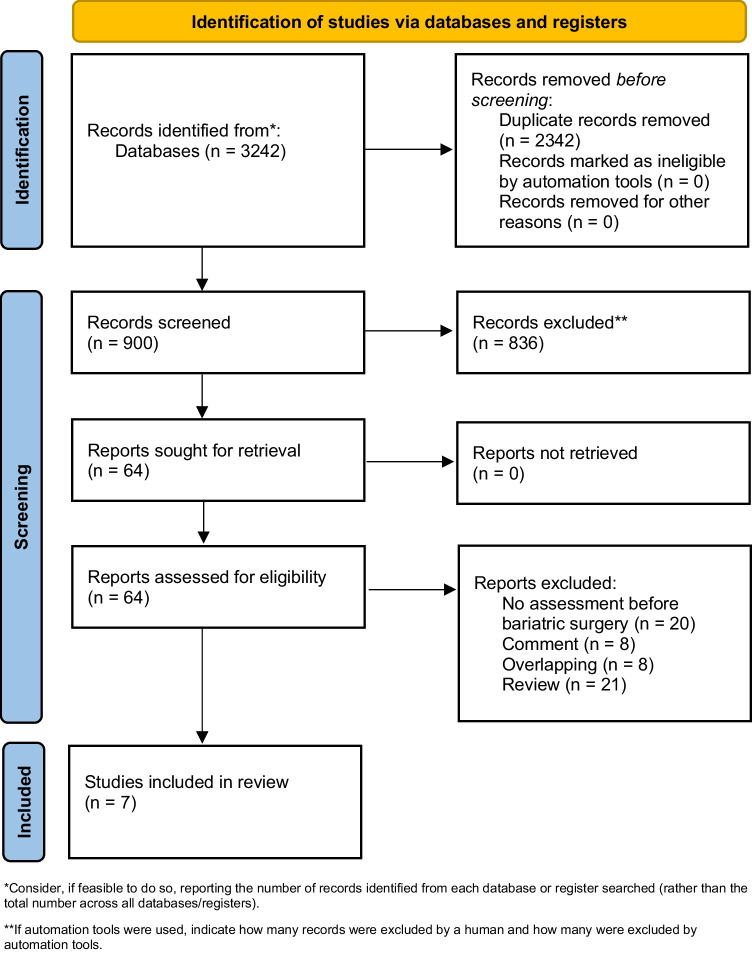
Flowchart according to PRISMA guidelines

A total of 3242 studies were retrieved, and 880 unique results remained for the initial title and abstract screening. Results were screened and 44 manuscripts underwent full-text review. Finally, only 7 articles met full inclusion criteria (Fig. 1). Studies included 3 retrospective cohort studies and 4 prospective cohort studies (Table 1).

**Table 1:** Design and characteristics of the included studies

References	Country	Inclusion period	Design	Purpose of the study	Nature and size of the sample	Measurement tool
Raebel, 2014	US	2005–2009	RCS	Evaluate SUDNO post-BS	10643 pre-BS no SUD, 421 SUDNO < 1yl post BS, 337 SUDNO > 1yl post BS	US Drug Enforcement Agency Schedule
King, 2017	US	2006–2009	PCS	Identify factors associated with opioids use after BS	2218 (RYGB 70.6%, AGB 24.9%, other 4.3%)	Self-reported survey
Smith, 2019	US	2006–2016	PCS	To establish prevalence of SUDNO after BS	27799 BS, 5976 pre-BS SUD, 21823 pre-BS no SUD of whom 1370 SUDNO	Self-reported survey
Wallen, 2023	Sweden	2007–2015	PCS	To compare SUD in BS patients VS obesity patients	Baseline 6 groups none/intermitted/chronic SUD in patients with a history of BS and none/intermitted/chronic SUD in obese patients	ICD-10
Butt, 2023	US	2018–2019	RCS	To establish rate of incidence NOSUD after BS and compare in 3 groups	Control cohorts: population with obesity (876720), general population (3532989), BS cohort (2523)	ICD-10
Svensson, 2023	Sweden	1987–2001	PCS	Investigate whether BS is associated with SUD	Control cohort: population with obesity (2037) BS cohort (2010)	ICD-10
Iranmanesh, 2023	Canada	2010–2021	RCS	Evaluate opioid use pre and post BS	11170 pre-BS no SUD, 263 SUDNO 1yl post-BS	Assessment of opioid use

*RCS* retrospective cohort study, *PCS* prospective cohort study, *AGB* adjustable gastric banding, *VBG* vertical banded gastroplasty, *GBP* gastric bypass, *RYGB* Roux en Y gastric bypass, *ORYGB* open Roux-en-Y gastric bypass, *RLYGB* laparoscopic Roux-en-Y gastric bypass, *LSG* laparoscopic sleeve gastrectomy, *BS* bariatric surgery, *SUD* non-alcoholic substance use disorder, *SUDNO* new-onset non-alcoholic substance use disorder

### Risk of Bias Assessment

By using the Risk of Bias in Non-randomized Studies of Interventions (ROBINS-I), there was low-to-moderate risk of bias among the included studies. Overall, 6 of the included studies had low risk of bias in the majority of items [23–28], while 1 of the included studies had some concerns for bias (moderate risk of bias in 5 items) [29]. None of the included studies was concerned to have a high risk of bias. Risk of bias assessment using the ROBINS-I tool is demonstrated in Table 2.

**Table 2:** Methodological quality evaluation of the included non-randomized studies according to ROBINS-I

Author	Bias due to confounding domains relevant to the setting of the study	Bias in selection of participants into the study	Bias in classification of interventions	Bias due to deviations from intended interventions	Bias due to missing data	Bias in measurement of outcomes	Bias in selection of the reported results
Raebel	Low	Low	Low	Low	Moderate	Low	Low
King	Low	Low	Low	Low	Moderate	Moderate	Low
Smith	Low	Low	Low	Low	Moderate	Moderate	Low
Wallen	Moderate	Moderate	Low	Low	Moderate	Moderate	Moderate
Butt	Low	Moderate	Low	Low	Moderate	Low	Moderate
Svensson	Low	Low	Low	Low	Low	Low	Moderate
Iranmanesh	Low	Moderate	Low	Low	Low	Moderate	Low

### Preoperative Patients’ Characteristics and Type of Surgery

Overall, 116,737 patients were included for analysis. Patient demographics from the included studies are displayed in Table 3. Female sex ranged from 70.7 to 83.6% in studies reporting sex. Mean age ranged from 40 to 47.6 in the included studies and mean BMI ranged from 40.5 to 48.9 kg/m^2^. Among the studies reporting the type of bariatric procedure performed, RYGB was most common ranging from 25.8 to 85.6%, whereas sleeve gastrectomy (SG) ranged from 2.2 to 61.2%.

**Table 3:** Preoperative patients’ characteristics and type of surgery

Author	Age	Female sex, %	BMI	Psychiatric comorbidities, %	University education, %	Type of surgery, *n* (%)
Raebel, 2014	47*	81.6	NA	13.3	12.8	SG = 234 (2.2) LRYGB = 6916 (65.0) ORYGB = 1147 (10.8) AGB = 1636 (15.4) Other = 710 (6.6)
King, 2017	46^	78.7	45.8^	9.9	36.8	RYGB = 1567 (70.6) AGB = 553 (24.9) Other = 98 (4.3)
Smith, 2019	NA	NA	NA	Anxiety = 60.9 Depression = 22.5 Bipolar = 41	NA	RYGB = 7622 (35) AGB = 2316 (10.6) SG = 11,683 (53.5)
Wallen, 2023	40*	79.4	40.5*	17	23.4	NA
Butt, 2023	47.6*#	76.4#	NA	NA	NA	SG = 1544 (61.2) RYGB = 652 (25.8) AGB = 327 (13)
Svensson, 2023	47.2*	70.7	42.4*	17	12.8	AGB = 373 (19) VBG = 1353 (68) RYGB = 264 (13)
Iranmanesh, 2023	45.7*	83.6	48.9*	42.9	NA	RYGB = 9567 (85.6) SG = 1591 (14.2) SADI = 8 (0.1) BPD-DS = 13 (0.1)

^*^Mean value

^Median value

*BMI*, body mass index; *LRYGB*, laparoscopic Roux-en-Y gastric bypass; *ORYGB*, open Roux-en-Y gastric bypass; *AGB*, adjustable gastric banding; *SG*, sleeve gastrectomy; *SADI*, single-anastomosis duodenoileostomy; *BPD-DS*, biliopancreatic diversion with duodenal switch

^#^Patients with SUDNO after BS

*NA*, not assessed

### Incidence of SUDNO and Predisposing Factors

Six of the 7 included studies evaluated the rate of SUDNO following bariatric surgery [23–28]. The retrospective cohort study by Butt et al. [26] reported an incidence of SUDNO as high as 6.55% after BS, which was consistent across other studies [23–25, 27, 28]. The lowest reported rate of SUDNO was 1.8% in the prospective cohort study by Svensson et al. [27]. A retrospective cohort study by Raebel et al. [23] determined rates of SUDNO up to 1 year following BS and demonstrated that post-surgery initiated SUD increased from 37.4 to 80% at 1-year follow-up. The incidence of substance abuse in the included studies was as follows: 40 cases/1000 people-year (Raebel et al. [23]), 116/1000 people-year (King et al. [24]), 62/1000 people-year (Smith et al. [25]), 33/1000 people-year (Butt et al. [26]), 0.8/1000 people-year (Svensson et al. [27]), 24/1000 people-year (Iranmanesh et al. [28].

**Fig. 2:**
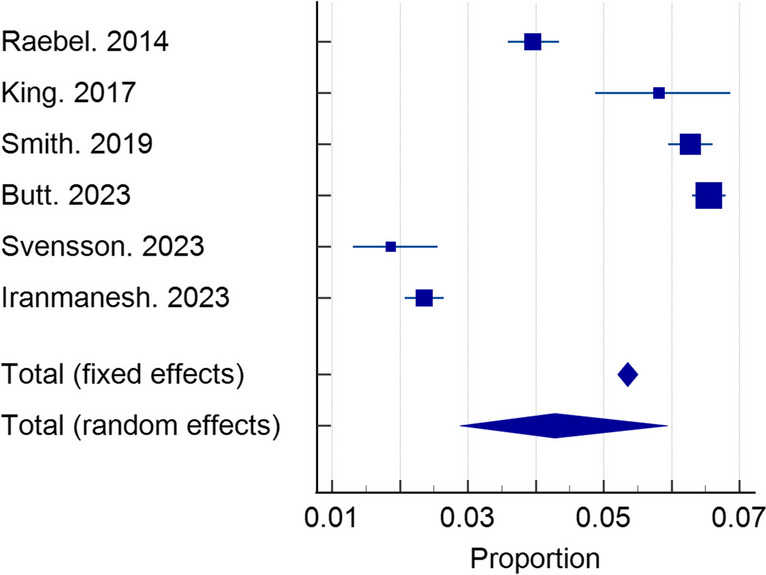
Pooled incidence of SUDNO after BS

The pooled incidence of SUDNO after elective bariatric surgery was 4.28% (*n* = 6 studies, 95% CI 2.88–5.94%, *I*^2^ = 99.07% random effects), as reported in Fig. 2. The follow-up periods and adherences to follow-up in the included studies are reported in Table 4.

**Table 4:** New-onset SUD rates and associated factors

Author	Patients included, *n*	Patients developing SUDNO, *n* (%)	Follow-up period and adherence to follow-up (%)	Patients’ factors associated with SUDNO	Surgical factors associated with SUDNO
Raebel, 2014	10643	421 (4.0)	1 year (NA)	Non-opioid analgesic use, younger age, mental illness	NA
King, 2017	2218	118 (5.8)	6 months (90.2%)	Public health insurance, pre-existing chronic pain	Postoperative chronic pain, reoperations, prescribed non-opioid analgesic use
Smith, 2019	21,823	1370 (6.3)	1 year (NA)	Caucasian, public health insurance, living alone, mental illness, metabolic comorbidities, higher pre-surgical BMI	Postoperative complications
Wallen, 2023	30,359	NA	2–8 years (NA)	NA	NA
Butt, 2023	38,525	2523 (6.55)	2 years (NA)	NA	NA
Svensson, 2023	1990	37 (1.8)	23.8 years (median) (NA)	NA	NA
Iranmanesh, 2023	11,179	263 (2.3)	1 year (60%)	Mental illness, metabolic comorbidities	Prescribed non-opioid analgesic use
Tot	116,737	4743	/	/	/

*SUDNO* non-alcoholic substance use disorder, *NA* not assessed, *BMI* body mass index

Four studies provided insight into secondary outcomes evaluating patients and surgical factors associated with SUDNO [23–25, 28]. Four studies demonstrated an association between SUDNO and pre-existing mental health disorders in patients undergoing BS [23–25, 28], and 3 demonstrated some surgical factors associated with SUDNO post-BS [24, 25, 28], as reported in Table 5. Severe complications post-surgery were also associated with SUDNO [24, 25]. Patient characteristics associated with SUDNO included preoperative mental illness, public health insurance coverage, and metabolic comorbidities (Table 4).

**Table 5:** Types of SUDNO

	Opioid	Cannabis	Nicotine	Hypnotics	Hallucinogens	Caffeine	Inhalants	Cocaine	Other
Raebel, 2014	X	NA	NA	NA	NA	NA	NA	NA	NA
King, 2017	X	NA	NA	NA	NA	NA	NA	NA	NA
Smith, 2019	X	NA	NA	NA	NA	NA	NA	NA	NA
Wallen, 2023	X	NA	NA	NA	NA	NA	NA	NA	NA
Butt, 2023	X	X	X	NA	NA	NA	NA	NA	X
Svensson, 2023	X	X	NA	X	X	NA	X	X	X
Iranmanesh, 2023	X	NA	NA	NA	NA	NA	NA	NA	NA

^#^Events related to SUD; *NA* not assessed

Surgical factors linked with SUDNO included severe postoperative complications and postoperative chronic pain and prescribed non-opioid analgesic use (Table 4).

### Types of SUDNO After BS

Among those studies reporting the developed type of SUDNO, chronic opioid use was the most common in 7 studies ranging from 22.3 to 100% of patients, compared to cocaine use (reported by 1 study in 3.6% of patients), cannabinoid use (2 studies; range 0–14%), hypnotics/sedative abuse (1 studies in 27% of patients), hallucinogens use (1 study in 1.8% of patients), as reported in Table 5.

## Discussion

The present study demonstrates a pooled incidence of SUDNO as high as 4.28%. The incidence varied across the included studies from 0.8 to 116/1000 people-year. The range of incidence in the included studies was wide; however, in 5 out of 6 studies reporting the data, the incidence was 24/1000 people-year or more. The lowest incidence was detected in the Swedish study; it should be highlighted that the inclusion period of the study started in 1987 and ended in 2001, whereas all the other included studies started including patients from 2005 or later. The higher incidence, on the other side, was detected in the studies from the USA. Furthermore, the method of detection of substance use differed, and studies based on self-reported survey had the higher incidence of SUDNO.

Patients undergoing bariatric surgery generally face a long journey before surgery. At first, they take the important decision to consult an obesity specialist. Then, they undergo multiple examinations and are in the care of a psychologist and a nutritionist with the aim of modifying the way of eating and generally the relationship with food before surgery to embrace a healthier lifestyle and maximize the beneficial effects of bariatric surgery. If we compare the incidence of substance use in the present review with the latest epidemiological data from USA, the latter appears to be higher: 46.3 million people aged 12 or older (or 16.5 percent of the population) met the applicable DSM-5 criteria for having a substance use disorder in the past year, including 29.5 million people who were classified as having an alcohol use disorder and 24 million people who were classified as having a drug use disorder [30]. However, the problem of substance abuse, even if it affects bariatric patients to a lesser extent than the general population (in the USA), is very relevant as this category of patients is fragile for many reasons. The finding of a cumulative incidence of 4.28% is important as it highlights the need of identifying patients at high risk of developing SUDNO, as they may have bad results after surgery and develop even lethal complications due to substance abuse, in consideration of the modified anatomy and the increased speed of absorption of psychoactive substances.

Second, the present study identifies some factors that predispose to SUDNO after bariatric surgery. Some of them are not modifiable (for example, level of education, age) but can allow us to select categories at higher risk, which could benefit from multidisciplinary management (psychiatric, psychological, psychosocial specialists) after surgery in order to minimize the occurrence of substance abuse.

Others (psychiatric illness, pre-surgical BMI, post-operative prescribed drugs, post-operative complications) can be treated/stabilized in the pre-operative period or are preventable or modifiable. Therefore, multidisciplinary assessment before surgery remains crucial before bariatric surgery, and in relation to SUDNO, it is important to know which patients are at risk and how to act to reduce their risk of developing SUD.

Concerning the relationship between obesity and substance abuse disorders, a large body of literature suggests that exposure to highly palatable food augments key neuroendocrine signals, which remodel the brain’s reward circuitry to reinforce pathological feeding behaviors [11, 31–33]. Similar to drugs of abuse, particularly palatable foods interact with brain reward circuits to promote their intake [34]. Interestingly, neurobiological features typical of addiction are observed in the brains of rats with obesity, deriving behaviors similar to addiction to foods rich in fat and sugar [34, 35], and suggesting an at least partially overlapping neurological basis. Consequently, if we postulate the presence of addiction pathology in some bariatric patients, it follows that these individuals might be vulnerable to the development of new substance use disorders addictions after the attenuation of food-derived reward produced by surgery [34]. This is supported by literature from patients undergoing bariatric surgery and placed in a substance abuse treatment program, 83% of whom identified “addiction substitution” in the etiology of addiction disorder [34, 36]. Note that in addition to this, some patients will also try to “fill the void” left by highly palatable food with another addiction, using the new substance abuse use as a coping strategy in response to stress or other negative emotions [34, 37].

Highly processed foods may present similar pharmacokinetic properties with drugs of abuse, i.e., concentrated dose and rapid rate of absorption, due to the addition of fat and/or refined carbohydrates [38]. Hyperpalatable foods and drugs of abuse may induce similar behavioral consequences, like craving, continuous use despite negative effects over own health, and reduced control over consumption [34, 39]. Reduced D2 receptor availability in obesity and substance use disorder vs. healthy controls may explain a dopamine deficiency in these patients [34, 40]. Food addiction, in a similar manner to drugs of abuse, has been supposed to decrease D2 receptors density [34].

Diet modulates endogenous opioids and cannabinoids as a function of palatability and causes delayed increases in dopamine by increasing glucose and insulin [41]. Industrial foods may exhibit pharmacokinetic properties similar to those of drugs of abuse, i.e., concentrated dose and rapid absorption rate, due to the addition of refined fats and/or carbohydrates [38], inducing behavioral consequences such as craving, reduced control over consumption, and continued use despite adverse effects on one’s health [34, 39]. The reduced availability of D2 receptors in obesity and substance use disorders compared with healthy controls may explain a dopamine deficiency in these patients [40]. In support of the positive impact of opioidergic neurotransmission in regulating food intake, a drug has long been approved for the treatment of obesity consisting of the combination of naltrexone and bupropion [34, 42].

Finally, it is evident that the anatomical change post-BS is able to alter the way in which the gastrointestinal tract communicates with the mesolimbic circuits and that this process participates in the control of appetitive aspects [11]. Recent studies highlight that neuroendocrine signals, such as GLP-1 and ghrelin, are potential signaling mechanisms that facilitate gastrointestinal communication with the mesolimbic dopaminergic system. Understanding how the brain adapts to gastrointestinal anatomical change continues to be an important area of investigation for obesity researchers and neuroscientists working on addictive disorders [11].

Surely, the present review highlights a real problem, substance use other than alcohol after bariatric surgery, giving some interesting data. The methodology was rigorous, as the literature was systematically reviewed, and the pooling of the data has been done to present a cumulative figure that can better photograph the extent of this disease. However, some limitations exist.

### Limits

The main limitation of this study is the nature and scarcity of the included studies. Three of the included studies are retrospective studies, and the data extracted from these papers are pooled with those provided by prospective studies. However, data from retrospective studies are extracted from prospective databases. By its nature, the pooling of information from multiple studies has limitations due to the significant variability in patient populations and study designs, as well as different pre-surgical assessment methods. Several bariatric procedures were included in this study, increasing the heterogeneity of the patient population. Furthermore, the studies were conducted in different health care system contexts. Prospective studies had limited size and numbers and were at risk for inclusion of nonconsecutive patients and nonresponse bias. Self-reported questionnaires were subject to recall bias or inaccurate responses regarding new psychoactive substance use due to stigma. Most of the included studies had chronic opioid use as their focus while there were scarce data on SUDNO from other substances of abuse. Due to the limitations of the data collected, we were unable to draw a comparison of the onset of SUDNO with control groups of obese patients who did not receive BS. Furthermore, the precise diagnosis of the type of associated mental disorder, which would be useful to further describe the impact of the development of new substance abuse disorders in these patients, is rarely specified in the included studies. Lastly, in 3 studies, the substance abuse was self-reported (2 studies) or assessed without clear mention of the assessment tool.

## Conclusions

The incidence of SUDNOs is a relevant complication after BS, ranging from 0.8 to 116/1000 people-year. Risk factors include patient-related factors, as previous mental illness, younger age, and use of public health insurance, and surgical factors, including postoperative complications and chronic pain. Preventive actions to reduce the occurrence of new SUDNOs in high-risk bariatric population are recommended.
